# Hyperpigmentation From Chronic Kratom Use: A Case Report and Review of the Literature

**DOI:** 10.7759/cureus.79804

**Published:** 2025-02-27

**Authors:** Bryan Tassavor, Chae Young Eun, Olga Nikolskaia, Ruth Jobarteh-Williams

**Affiliations:** 1 Medical School, A.T. Still University School of Osteopathic Medicine in Arizona, Mesa, USA; 2 Medical School, Drexel University, Philadelphia, USA; 3 Department of Pathology, Drexel University, Philadelphia, USA; 4 Department of Dermatology, WellSpan Health, York, USA

**Keywords:** cutaneous hyperpigmentation, drug-induced hyperpigmentation, kratom addiction, kratom toxicity, photodistributed hyperpigmentation

## Abstract

Kratom, an opioid substitute derived from the leaves of *Mitragyna speciosa*, is commonly used for its opioid-like effects in East Asia and is gaining popularity in the United States. A rare side effect of chronic kratom use, previously noted in the literature, is skin hyperpigmentation. We present a unique case of diffuse, photodistributed hyperpigmentation in a patient with over a decade of kratom use. This case is accompanied by an evaluation of the adverse effect, focusing on its clinical presentation, histopathological findings, and potential mechanism. Additionally, we provide a brief literature review on the topic.

## Introduction

Kratom is a psychoactive substance extracted from the leaves of the *Mitragyna speciosa *tree that is often used as an inexpensive opioid substitute in the form of powder or tea leaves. Kratom formulations include the alkaloid compounds mitragynine and 7-hydroxymitragynine (7-OHMG), which are theorized to primarily act as partial agonists at μ-opioid receptors while also activating dopaminergic receptors [1]. Kratom has recently become more commonly used in the United States, with data collected between 2018 and 2021 suggesting a yearly prevalence of two million active users and three million lifetime users [2]. A rare cutaneous side effect that has recently been associated with kratom is photodistributed hyperpigmentation. We report another rare case of hyperpigmentation associated with chronic kratom use and review what is currently known about this adverse effect of the substance.

## Case presentation

**Figure 1:**
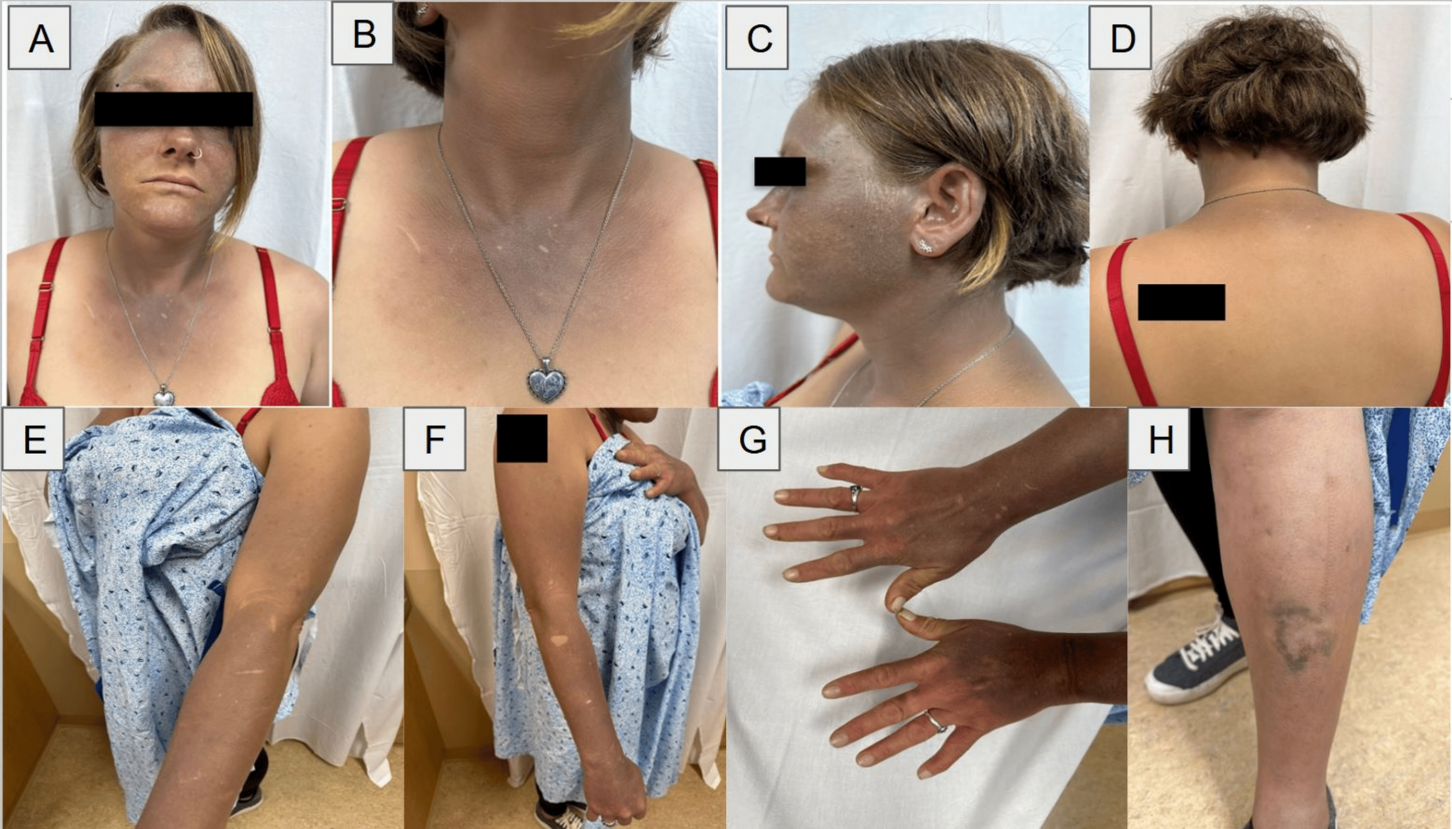
Kratom-induced hyperpigmentation at clinic visit. (A) Frontal view showing hyperpigmentation of the face, neck, and chest. (B) Frontal view highlighting hyperpigmentation of the neck and chest. (C) Left profile view displaying hyperpigmentation of the face and neck. (D) Dorsal view showing hyperpigmentation of the neck. (E) Left side view demonstrating hyperpigmentation of the arm and forearm. (F) Right side view showing hyperpigmentation of the arm and forearm with sparing of old scars. (G) Top view of both hands showing knuckle-sparing hyperpigmentation. (H) Side view of the left shin displaying hyperpigmentation surrounding an old scar.

A 34-year-old Caucasian female patient presented to the dermatology clinic two years ago for hyperpigmentation, which started six years prior. Physical examination revealed gray-blue patches in a photodistributed pattern involving her face, chest, arms, and hands with knuckle sparing (Figure 1A-1G). There was also a similar pigmentary change around the edges of an old scar on her left shin (Figure 1H). Her medical history was significant for levetiracetam use for seizure management, untreated hepatitis C, and multiple periods of intravenous drug use, last used in 2016. Bloodwork ruled out hemochromatosis, human immunodeficiency virus (HIV), heavy metal consumption, and Addison's disease. Classical drug-induced hyperpigmentation was considered, but the patient had denied a history of commonly implicated medications like minocycline and amiodarone. A shave biopsy of her right forearm was performed, revealing findings suggestive of drug-induced hyperpigmentation. Of note, the patient also denied the use of hydroquinone so drug-induced ochronosis was not suspected. Levetiracetam was subsequently suspected to be the cause of the unlikely drug-induced hyperpigmentation due to supporting literature indicating it as a possibility [3]. The drug was subsequently discontinued.

**Figure 2:**
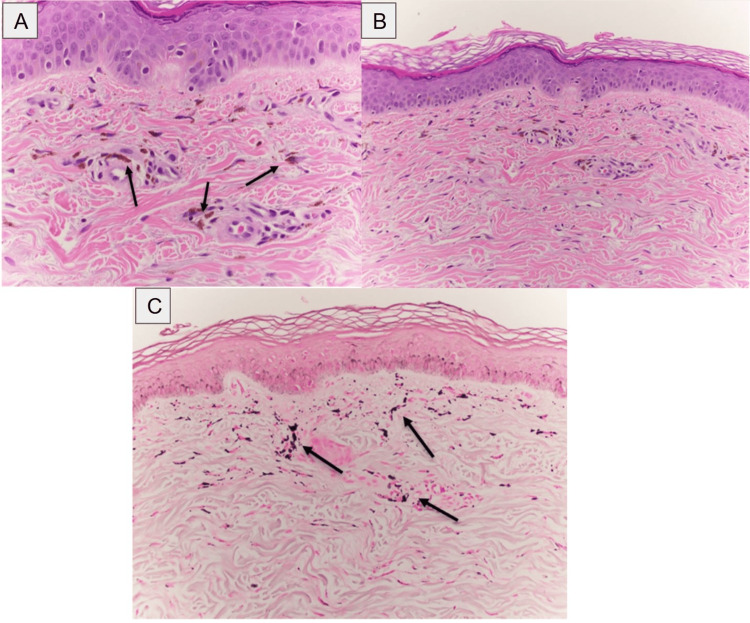
Kratom-induced hyperpigmentation skin biopsy. (A) Hematoxylin and eosin stain was used to identify the possible distribution of pigment at the skin biopsy sites. The biopsy depicts evidence of melanin incontinence as evidenced by the arrows with the superficial dermis showing deposits of pigment. (B) Hematoxylin and eosin stain was used to identify the architecture of the dermis and epidermis at the skin biopsy sites. This biopsy depicts unchanged epidermal architecture. (C) Fontana-Masson stain was used to identify pigment incontinence at the site of the skin biopsy as indicated by the arrows.

Despite discontinuation, the hyperpigmentation continued to worsen by the current visit this year. Upon further questioning, the patient mentioned that she was a frequent drinker of kratom tea as an opioid substitute and had been using it for over 12 years. The patient had been taking 6-8 teaspoons of kratom daily in a paste formulation. The biopsy from the initial visit, where histological sections from the patient's upper forearm were taken, was re-examined with further histochemical stains. The sections showed numerous superficial dermal deposits of clustered brick brown, nonpolarizable pigments, ranging in size from 1µm to 2 µm (Figure 2A). In addition, several isolated linear clumps of pigments were found parallel to the dermal collagen fibers. The epidermal architecture was maintained with the preserved normal distribution of junctional melanocytes (Figure 2B). There was no background inflammation or other morphological features suggesting inflammatory dermatosis. CD117 immunostaining did not reveal an increased number of mast cells in the superficial dermis. Prussian blue iron stain was negative for hemosiderin; however, the pigments were positive for the Fontana-Masson stain, a melanin marker (Figure 2C).

## Discussion

Previously published literature has implicated kratom use as a cause of hyperpigmentation, making it our favored causative agent over levetiracetam given that the pigmentation persisted despite the discontinuation of the latter (Table 1) [4-14]. The combined morphological findings in our biopsy results, together with clinical history, were suggestive of kratom-induced hyperpigmentation. Differential diagnosis could include another variant of drug-induced hyperpigmentation or argyria; however, due to clinical history and distinct morphological features of the pigment deposits, a diagnosis of kratom-induced pigmentation was made.

**Table 1:** A chronological summary of the findings in the relevant literature associating kratom with hyperpigmentation. Study findings were organized by patient demographics, kratom regimen and ROA, length of kratom use, details regarding the distribution of hyperpigmentation, and outcome regarding kratom use. NR: not reported; apx: approximately; 7-OHMG: 7-hydroxymitragynine; ROA: route of administration

Study authors (publication year)	Age (years), sex, race of patient(s)	Kratom ROA, dose, regimen	Length of kratom use	Location and description of pigmentation	Final outcome
Suwanlert (1975) [4]	30 Thai participants with one described: 55 years, male, Thai	Chewing leaf or consumption of ground leaf with hot fluids. Participants in the study consumed 10-30 leaves a day	1-35 years; the patient described had been using kratom for 35 years	Darkening complexion on the face and cheeks bilaterally	NR; only one participant is open to discontinuing kratom
Tunsuriyawong (2002) [5]	66 years, male, Thai	Chewing leaf. The patient chewed 25 kratom leaves a day	30 years with pigmentation beginning in the last 5-6 years	Darkening of the skin on the trunk, back, and extremities with buttock and skin crease sparing	NR
Vicknasingam et al. (2010) [6]	132 Malaysian participants, 3 Chinese participants, and 1 "Indian/Other" participant	Fluid preparation. Participants consumed 3.2 glasses of kratom daily on average (apx 250 ml per glass)	72 participants had been using for 2 years or less, while 64 patients had been using for over 2 years	Hyperpigmentation of cheeks was reported in 42 short-term users and 21 long-term users	NR; 78% of participants reported being unable to cease use of kratom
Saingam et al. (2013) [7]	34 male Thai participants	Chewing leaf. Chronic users had consumed 10-80 leaves per day on average, while sporadic users had consumed 1-20 leaves per day on average	Chronic users had been using kratom for 3-50 years continuously, while sporadic users had been using it for 1-6 years	The study reported that "regular users" in the study had “dark skin"	NR; 18 users unsuccessfully tried to quit kratom use, while 3 users quit successfully. No mention of change in pigmentation
Eaimchaloay et al. (2019) [8]	106 Thai participants with an average age of 32.6 years	45 users chewed kratom leaves, 60 users boiled kratom, and 1 user consumed kratom capsules. 21 users reported using kratom less than 3 times a day, while 24 users reported using kratom more than 3 times a day	38 kratom users part of the chewing group reported having used kratom for more than a year, while 51 kratom users in the boiling group reported having used kratom for over a year	41.9% of total users reported hyperpigmentation as an adverse result of kratom use	NR
Powell et al. (2022) [9]	54 years, male, Caucasian	Powdered form consumed with orange juice 3-4 times a day	4-5 years	Blue-to-gray hyperpigmentation in patches on the arms and face	NR
Johnson et al. (2023) [10]	56 years, female, NR	4-5 doses (unspecified) per day	7 years	Blue-to-gray hyperpigmented patches on the face, neck, chest, arms, and legs	NR
Suleman et al. (2023) [11]	32 years, male, Caucasian	Kratom supplements (unspecified)	4-5 years	Blue-to-gray hyperpigmented patches over the hands (sparing knuckles), arms, face, and neck	NR
Patel and Phelan (2024) [12]	30 years, male, Caucasian	Kratom capsules, 815 grams daily for the first year and 3-7 grams daily for the next 4 years	5 years (hyperpigmentation began 4.5 years into use)	Dark gray-to-blue hyperpigmentation of the cheeks of the face, back of the neck, and backs of the hands and forearms	Hyperpigmentation has not regressed in the 16 months after discontinuing kratom
Gandhi et al. (2024) [13]	63 years, male, NR	3 bottles of liquid kratom per day (apx 180 mg of mitragynine and less than 8 mg of 7-OHMG)	5 years (hyperpigmentation began 4 years into use)	Tender and pruritic hyperpigmented patches on the face, neck, and forearm	NR
Tassavor et al. (2024)	34 years, female, Caucasian	6-8 teaspoons of kratom daily in a paste formulation	12 years (hyperpigmentation began 6 years into use)	Brown-to-gray hyperpigmented patches on the face, chest, arms, hands (with knuckle sparing), and left shin	NR

In the earliest of the referenced studies, Suwanlert described the observation that chronic kratom users with substance abuse disorders can present with a muddy gray complexion akin to those seen in cirrhotic patients. This general finding of skin hyperpigmentation among long- and short-term kratom users was also referenced in several survey studies in eastern countries, summarized in Table 1 [4,6-8]. In the referenced case reports, the studies describe patients who presented with a photodistributed pattern of pigmentation following kratom use akin to our patient's presentation [5,9-13]. The patient presented in this report has a darker and more diffuse photodistributed pattern of hyperpigmentation, similar to the patient in Tunsuriyawong's study [5]. This highlights the possible progressive nature of this adverse effect. This patient also featured knuckle sparing similar to the patient in Suleman et al., indicating a possible recurring clinical pattern with this adverse effect [11]. A unique pigmentation pattern seen in this study is pigmentation surrounding old scarring. This has not been seen in the referenced literature.

In Tunsuriyawong, the earliest published case report, a biopsy revealed epidermal hyperpigmentation and numerous melanophages in the papillary dermis [5]. In Powell et al., hand and elbow biopsy sections revealed a normal distribution of junctional melanocytes, scattered deposits of nonpolarizable intrahistiocytic, perivascular, and interstitial red-brown pigment, and a positive Fontana-Masson stain [9]. The biopsy results were also able to rule out the presence of inflammation, hemosiderin, fungal or bacterial infection, and pigmented purpuric dermatosis. Our study had similar findings regarding the distribution of junctional melanocytes, lack of evidence for inflammation, and hemosiderin. However, the deposits of pigments in our study had a smaller distribution of sizes, with some deposits appearing parallel to collagen fibers. The positive Fontana-Masson stain in both biopsy results and the absence of minocycline or amiodarone in either patient give credence to the notion that kratom may be involved in inducing melanin production. In Johnson et al., neck biopsy results showed pigment-laden histiocytes with a negative result on Fontana-Masson and Prussian blue stains [10]. In Suleman et al., a biopsy revealed deposits of refractile, perivascular intrahistiocytic brown pigment [11]. The pigment deposition stained positive with Fontana-Masson stain and negative for periodic acid-Schiff and Prussian blue stains. This study supports our and Powell et al.'s conclusions, given its positive Fontana-Masson stain [9]. Together, these findings suggest biopsy and histochemical staining are reliable diagnostic tools for evaluating kratom-induced hyperpigmentation.

To this point, the mechanism behind kratom's ability to cause hyperpigmentation is largely unclear. It has been postulated that mitragynine can cause the activation of melanocyte-stimulating substances via the modulation of D2 receptors [14]. This, in turn, may be linked to the activation of the melanocyte-stimulating substances. Additionally, it is unclear what the exact formulation of the kratom consumed by the patient was, leaving open the possibility of trace elements that may have played a role as well. 

## Conclusions

Our report presents a unique case of kratom-induced hyperpigmentation in a chronic user characterized by knuckle sparing and scar pigmentation. Although the underlying mechanism remains speculative, our findings suggest that histochemical staining may be reliable for assessing this adverse effect. A review of the literature indicates that this reaction has been documented in survey studies in East Asia and is now emerging as a concern in the United States. As kratom use rises, it is crucial for dermatologists to recognize hyperpigmentation as a potential sign of kratom toxicity.
